# Broad-Spectrum Antiviral Strategies and Nucleoside Analogues

**DOI:** 10.3390/v13040667

**Published:** 2021-04-13

**Authors:** Robert J. Geraghty, Matthew T. Aliota, Laurent F. Bonnac

**Affiliations:** 1 Center for Drug Design, College of Pharmacy, University of Minnesota, Minneapolis, MN 55455, USA; gerag012@umn.edu; 2 Department of Veterinary and Biomedical Sciences, University of Minnesota, St. Paul, MN 55108, USA; mtaliota@umn.edu

**Keywords:** broad-spectrum antivirals, lethal mutagenesis, chain terminator, nucleoside analogues

## Abstract

The emergence or re-emergence of viruses with epidemic and/or pandemic potential, such as Ebola, Zika, Middle East Respiratory Syndrome (MERS-CoV), Severe Acute Respiratory Syndrome Coronavirus 1 and 2 (SARS and SARS-CoV-2) viruses, or new strains of influenza represents significant human health threats due to the absence of available treatments. Vaccines represent a key answer to control these viruses. However, in the case of a public health emergency, vaccine development, safety, and partial efficacy concerns may hinder their prompt deployment. Thus, developing broad-spectrum antiviral molecules for a fast response is essential to face an outbreak crisis as well as for bioweapon countermeasures. So far, broad-spectrum antivirals include two main categories: the family of drugs targeting the host-cell machinery essential for virus infection and replication, and the family of drugs directly targeting viruses. Among the molecules directly targeting viruses, nucleoside analogues form an essential class of broad-spectrum antiviral drugs. In this review, we will discuss the interest for broad-spectrum antiviral strategies and their limitations, with an emphasis on virus-targeted, broad-spectrum, antiviral nucleoside analogues and their mechanisms of action.

## 1. The Interest of Broad-Spectrum Antiviral Strategies

**Figure 1:**
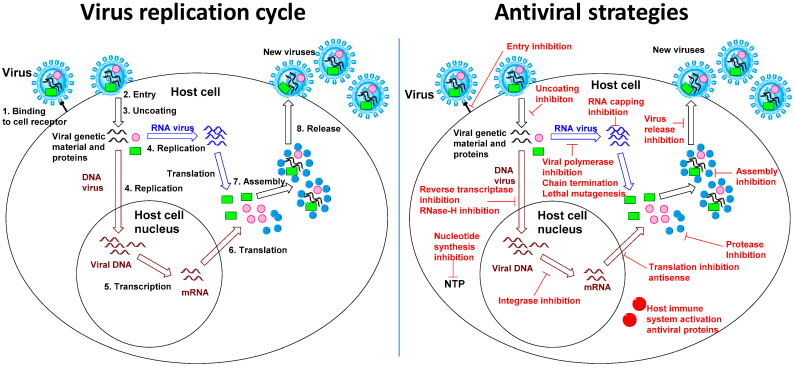
Virus replication cycle and antiviral strategies.

Antiviral drug development has long focused on virus-specific approaches: the strategy to study a virus and identify a specific viral protein as a drug target in order to limit potential toxicity and to increase drug efficacy (Figure 1). Virus-specific drug development has also been favored as a way to simplify the drug discovery process compared to the more complex design of broad-spectrum antivirals, which often require targeting critical proteins belonging to different viruses or critical cellular processes used by different viruses. Virus-specific antiviral research remains a successful and essential strategy to combat viral infections [1]. However, a major obstacle to virus-specific drug development is the length of the process, which, on average, takes more than a decade between research and drug approval [2]. Indeed, virus-specific drug development needs first to address fundamental biology of the virus to find an appropriate target before moving to medicinal chemistry research, compounds screening, lead optimization, animal studies and eventual clinical trials. The time constraint for antiviral drug discovery has gradually become more important due to the increasing number of viral outbreaks that have recently and consecutively afflicted our societies including Ebola virus [3], Middle East Respiratory Syndrome coronavirus MERS-CoV [4], Severe Acute Respiratory Syndrome Coronavirus 1 and 2 (SARS and SARS-CoV-2) [5,6], new strains of influenza A virus [7] as well as dengue virus [8], Zika virus [9,10], West Nile virus [11] and Chikungunya virus [12]. Reasons for the increased appearances of these outbreaks include increased population densities, forest fragmentation, intercontinental traveling, and climate change [13,14]. Regardless of their origins, the recent and consecutive nature of these outbreaks highlight the challenges of virus-specific drug research for fast discovery of a therapeutic. Similarly, vaccine development, although particularly relevant to face outbreaks and pandemics, also requires a significant development time to ensure the efficacy and safety of the vaccine [15]. Re-purposing antiviral drugs, the strategy to evaluate approved drugs for known viruses against new viruses is highly advantageous in terms of reducing cost and saving time, and has therefore been an essential component of emergency responses during public health crisis [16].

Drug re-purposing is advantageous in that the initial part of the drug discovery process can be bypassed including medicinal chemistry research and lead optimization as well as the safety evaluation in phase 1 clinical trials, therefore reducing considerably the research and development time. Yet, re-purposing antiviral drugs successfully is inherently dependent on the ability of an antiviral drug to be active against multiple viruses, hence, the need of a broad-spectrum antiviral effect. Historically, broad-spectrum antivirals have first been discovered by serendipity through simple screening assays against different viruses rather than through a deliberate strategy to design drugs to display a broad-spectrum antiviral effect [17,18] for ribavirin or more recently with antibiotic derivatives with antiviral properties [19,20]. From these initial antiviral drug evaluations emerged patterns of drugs with similar biological effects. Broad-spectrum antiviral drugs can be classified into two main categories; first, drugs that affect the host-cell machinery essential for the infection and replication of different viruses, and second; drugs targeting viruses directly. Both families of broad-spectrum antiviral drugs (e.g., host-targeted or virus-targeted broad-spectrum antivirals) present advantages, and disadvantages (Table 1).

## 2. Host-Targeted Antiviral Strategies

Host-targeted antiviral strategies, reviewed here [21,22], feature a wide range of host targets including host protease inhibition to restrict viral entry, depletion of intracellular nucleotide pools, kinase inhibition, glycosidase inhibition, immune system activation and more. While the main disadvantage of host-targeted antivirals is the higher risk for host toxicity, an advantage is that the host-targets/proteins involved in virus replication are often known and can be studied before a new virus emerges.

A host-targeted approach often offers a higher barrier to the appearance of viral drug resistance. In practice, developing both host-targeted and virus-targeted broad-spectrum antivirals is crucial and complementary to answer the viral outbreaks to come. Virus-targeted- broad-spectrum antiviral strategies present their own challenges; the diversity of viral protein structures and sequences make the design of broadly acting compounds particularly difficult, and often result in a limited spectrum of antiviral applications. Up until now, only one class of compounds, nucleoside analogues, has shown promise for broad-spectrum antiviral applications in the clinic. Viruses adopt different replication strategies, yet all replicate their genetic material via a DNA or RNA polymerase conferring potential susceptibility to molecules resembling the natural nucleoside building blocks of their genomes; the antiviral nucleoside analogues. Many viruses also share the ability to adapt rapidly to new stress conditions which makes them vulnerable to antiviral strategies targeting their mutation frequency.

## 3. Broad-Spectrum Antiviral Nucleoside Analogues

**Figure 2:**
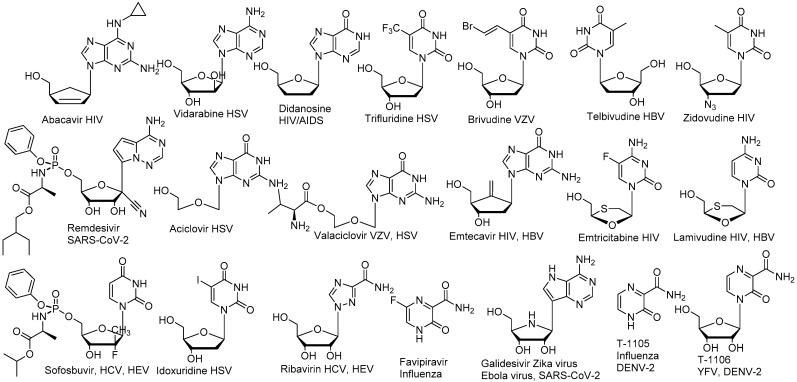
Non-exhaustive list of nucleoside antiviral drugs and other antiviral nucleoside analogues.

Antiviral nucleoside research began in the 1960–1970s [18,23] but demonstrated its enormous potential in the 1980–1990s with the discovery of several anti-HIV drugs such as Abacavir, AZT and other drugs reviewed here [24,25] and since then has expanded its applications to other viral pathogens (Figure 2) [26,27,28]. Many direct-acting antiviral nucleoside analogues target the viral polymerase responsible for the viral genome replication [29]. Unlike non-nucleoside polymerase inhibitors that often bind to allosteric non-conserved and -mutation-tolerant sites, nucleoside analogues directly bind to the more conserved active site of the viral polymerase after conversion to their triphosphate active form [30].

Although antiviral nucleoside analogues form a major class of antiviral drugs (Figure 2), the development of nucleoside antiviral inhibitors faces several challenges. Nucleoside analogue half-life and pharmacokinetics differ significantly from natural nucleoside properties [30]. Many of these challenges originate from the very nature of nucleoside inhibitors that intrinsically require activation by host kinases to their triphosphate active form before becoming substrate of the viral polymerase [31]. The efficiency of antiviral nucleoside triphosphate formation varies significantly from one cell type to another due to the different levels of kinases required for the phosphorylation process, the antiviral nucleoside triphosphate level in cells being recognized as a good indicator for the antiviral activity [1,32]. While the second phosphorylation can be a critical step [33,34], the first phosphorylation is often the rate-limiting step for triphosphate formation for most nucleoside analogues [30,35]. Therefore, nucleoside monophosphate prodrugs have been developed successfully to bypass the first phosphorylation issue and result in improved antiviral efficacies [36,37,38]. This is exemplified by the successful development of the anti-HCV drug Sofosbuvir, a nucleoside phosphoramidate prodrug [1,32] following initially underwhelming studies of poorly active nucleoside analogues [32,39]. The nucleoside phosphoramidate prodrug is able to enter infected cells where it is directly converted to the monophosphate therefore bypassing the first phosphorylation issue.

The definition of “broad-spectrum antiviral” can be considered a matter of scale, with some defining the term to encompass all viruses, or perhaps all RNA viruses, or some defining more narrowly to include all viruses of a particular family. The number of direct-acting and virus-targeted nucleoside analogues displaying an antiviral effect across different virus families is limited. Three nucleoside analogues stand out as broad-spectrum RNA virus inhibitors: Remdesivir, Ribavirin and T-705/Favipiravir. We will further discuss these three nucleoside analogues, their antiviral activities, and mechanisms of action.

### 3.1. Remdesivir

**Figure 3:**
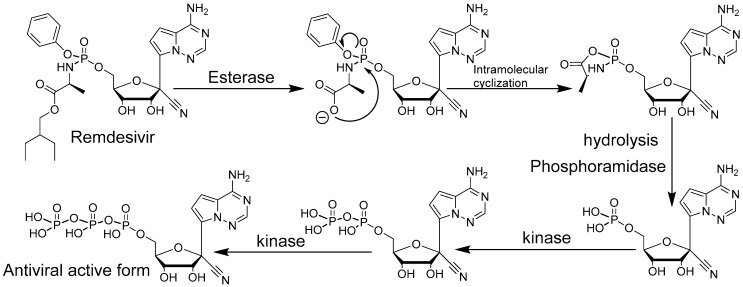
Remdesivir activation mechanism.

Remdesivir is a *C*-nucleoside analogue and a phosphoramidate prodrug that resembles adenosine monophosphate. The *C*-nucleoside nature of Remdesivir, with the replacement of the natural carbon–nitrogen bond between the base and the sugar, by a carbon–carbon bond, is advantageous in terms of chemical and enzymatic stability (Figure 3). The *C*-nucleoside structure also allows the unusual 1′ modification of the sugar. Nucleosides with a 1′ modifications are often poorly stable with the regular carbon–nitrogen bond between base and sugar and/or difficult to synthesize. The Ebola virus outbreak in West Africa in 2013 increased antiviral drug research and screening resulting in a selection of promising leads. Research centers started intensive studies, including in non-human primates, that lead to the identification of Remdesivir, a broad-spectrum antiviral active against Ebola and, Marburg viruses as well as against MERS-CoV and SARS-CoV-2 [40,41]. Remdesivir is an FDA-approved drug for the treatment of COVID-19 patients [42,43]. Remdesivir displays a remarkably broad antiviral effect [44,45] rare for molecules with similar mode of action through viral RNA chain termination. Before acting as a delayed chain terminator [46,47], the Remdesivir phosphate prodrug must enter infected cells where it is converted metabolically by different enzymes to its triphosphate active form (Figure 3).

Remdesivir, under its triphosphate active form then becomes substrate of the viral polymerase, a highly conserved and critical protein of the viral replication cycle [48,49]. Remdesivir triphosphate mimics the natural substrate nucleotide adenosine triphosphate. Delayed chain termination occurs 3-to-5 nucleotides after Remdesivir triphosphate is incorporated into a growing new strand of viral RNA genome [46,47] leading to a stalling mechanism [46,48,50]. It is worth noting that the delayed chain termination might protect Remdesivir from excision by viral proofreading proteins thanks to the 3–5 additional natural nucleotides [51]. The delayed chain termination triggered by Remdesivir forces the premature ending of the viral RNA synthesis. A downside of Remdesivir is the need to administer it intravenously [43,52], likely due to its poor oral bioavailability and short half-life [53]. Alternatives to Remdesivir or orally available formulations of Remdesivir are therefore needed to make treatment more practical.

### 3.2. Ribavirin

**Figure 4:**
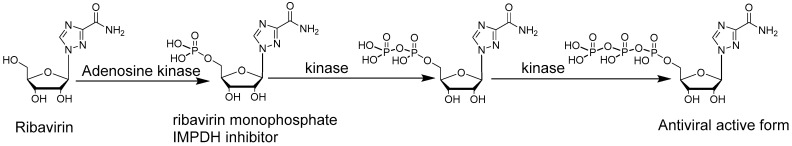
Ribavirin activation mechanism.

Ribavirin discovered in the 1970s is one of the most remarkable nucleoside antivirals due to its antiviral activities, both in tissue culture and animal models against a uniquely broad range of viruses including DNA and RNA viruses [17] (Figure 4). Ribavirin [18] is an approved drug against hepatitis C infection in combination with interferons alpha or in combination with other medications such as sofosbuvir [54,55]. Ribavirin has also been used for the treatment of other viral infections [56,57]. Structurally, ribavirin possesses a regular ribose moiety linked to a triazole aromatic ring as a base with a rotatable amido group attached to it. The rotatable amido group makes ribavirin resemble adenosine or guanosine upon its rotation. In cells, ribavirin nucleoside is phosphorylated by adenosine kinase to its monophosphate form and further processed by other kinases to its triphosphate form (Figure 4) [58]. The antiviral mechanism of action of Ribavirin is still under investigation and likely comprises multiple mechanisms [59]. These mechanisms include host-targeted effects such as the inhibition of inosine monophosphate dehydrogenase (IMPDH) under its 5′-monophosphate form and host immune response modulation, as well as virus-targeted effects such as viral polymerase inhibition, viral lethal mutagenesis, and viral RNA capping inhibition. The wide variety of viruses susceptible to ribavirin is likely due to the combined antiviral effects triggered by ribavirin [17]. For host-targeted antiviral effects, ribavirin monophosphate inhibits IMPDH [60] which is responsible for the conversion of inosine monophosphate (IMP) to xanthosine monophosphate (XMP). IMPDH role is crucial for DNA and RNA synthesis since IMPDH controls the intracellular guanine nucleotide (GTP and dGTP) pool concentrations which may explain the activity of ribavirin against both DNA and RNA viruses [17]. Ribavirin also possesses an immunomodulatory effect by modifying the host T-cell response through a switch in T-cell phenotypes [61]. Regarding virus targeted antiviral effects, ribavirin, as a guanosine analogue [62], can interact with the RNA capping enzymes [63]. Capped RNAs contains a 7-methylguanosine cap structure essential for RNA stability and translation. Inhibition of viral RNA capping is interesting in that the lack of viral RNA capping triggers the antiviral host immune response by recognition of a foreign viral RNA. Ribavirin also displays virus-targeted effects, with the inhibition of the viral RNA-dependent RNA polymerase (RdRp) and viral lethal mutagenesis. Ribavirin structure is quite different from typical RdRp chain terminators that usually display modifications on the sugar that prevent further elongation of the growing viral RNA. Ribavirin does not have modifications on its sugar moiety but only on its base, so it remains unclear how ribavirin inhibits RdRp. Yet ribavirin incorporation can significantly reduce RdRp catalytic efficiency of viral RNA synthesis. It has been demonstrated that ribavirin triphosphate inhibits the influenza A virus RNA polymerase in vitro [64] as well as hepatitis C virus RNA polymerase [65], vesicular stomatitis virus [66,67]. Finally, ribavirin also induces an antiviral effect via viral lethal mutagenesis against several viruses [68,69] including in vivo against hepatitis C [70]. Most RNA viruses possess a high mutation rate that allows them to adapt, escape host immune defenses and drug treatments [71,72]. The high error-prone nature of viral RdRps is widely recognized as the main source for the virus high mutation rate [73,74]. However, RNA viruses possess an error threshold above which genetic information cannot be maintained. Alteration of the viral mutation rate with ambiguous base-pairing nucleoside analogues has been proposed as a potential therapeutic approach, called lethal mutagenesis, targeting high-mutation-rate RNA viruses [75]. Ribavirin increases the viral RNA mutation frequency to non-viable levels thanks to its ambiguous base-pairing capacity. When incorporated into the viral RNA, ribavirin base-pairs equally with uridine or cytosine nucleotides consequently inducing viral mutations [68,69]. With the accumulation of deleterious mutations within its genome, the virus no longer maintains the genetic information required for survival, a process called lethal mutagenesis or error-catastrophe. The understudied family of compounds able to induce viral lethal mutagenesis offer a unique chance for broad-spectrum antiviral activity, theoretically able to affect most high-mutation-rate RNA viruses. Overall, ribavirin’s ability to mimic both adenosine and guanosine is advantageous in that it allows ribavirin to interact with a variety of enzymes and biological mechanisms critical for the replication cycle of many viruses. The downside of ribavirin resembling adenosine and guanosine is that it enhances the interaction of ribavirin with the host cell machinery resulting in poor selectivity and toxicity [76] yielding undesirable side effects such as severe anemia. Discovery of molecules with similar ambiguous base-pairing capacity but more selective for viral proteins is therefore desirable [77].

### 3.3. T-705 Favipiravir

**Figure 5:**
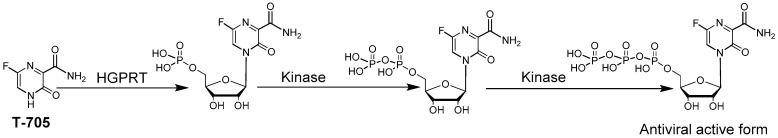
T-705/Favipiravir activation mechanism.

As mentioned in Section 3.2., compounds able to induce viral lethal mutagenesis offer a unique opportunity for broad-spectrum antiviral activities. Viral lethal mutagenesis is interesting because even a small increase in the viral mutation rate can have a devastating negative impact on virus replication. The lethal mutagenesis inducer ribavirin is broadly active yet poorly selective and toxic, thus alternatives are needed. An approved drug in Japan against influenza T-705 (Favipiravir/Avigan) (Figure 5) possesses a broad-spectrum antiviral effect [78,79], and can induce lethal mutagenesis of multiple viruses as well as chain termination. T-705 is a nucleobase analogue (the base of a nucleoside without ribose), a pyrazinamide derivative that resemble adenine or guanine upon rotation of its amido group [80]. In cells, T-705 nucleobase is converted enzymatically to its corresponding mononucleotide by the hypoxanthine-guanine phosphoribosyl-transferase (HGPRT) of the purine nucleotide salvage pathway [81]. Then T-705 mononucleotide is further phosphorylated to its triphosphate active form by different kinases (Figure 5) [82,83]. T-705 is interesting in that, as a nucleobase, it bypasses the first phosphorylation step that is often rate limiting for antiviral nucleosides. Instead, it is directly converted from nucleobase to mononucleotide by HGPRT [81]. Once converted to its nucleoside triphosphate form, T-705, like ribavirin, is incorporated by the viral RdRp as an ATP or GTP mimic to induce its antiviral effect via lethal mutagenesis [84] and/or chain termination [85,86]. Unlike ribavirin, T-705 appears to lack overt toxicity issues [77,78]. T-705 does not appear to inhibit host DNA or RNA polymerases [83,86]. Previous studies have also demonstrated that high concentrations of T-705 do not influence the synthesis of cellular RNA or DNA [82] and that T-705 does not cause mitochondrial toxicity [87]. In clinical trials, T-705 has been administered in gram amounts to patients [88,89] without significant side effects. T-705 mononucleotide is a weak inhibitor of IMPDH [82] and therefore does not affect endogenous nucleotide pools significantly. 

As mentioned earlier, T-705 induces viral lethal mutagenesis but T-705 can also display antiviral activity through viral RNA chain termination. These two antiviral effects, lethal mutagenesis and chain termination, appear antinomic since these mechanisms should exclude each other. However, a recent publication by Canard and colleagues seems to reconcile both mechanisms [80] by taking into consideration the ambiguous base-pairing nature of T-705. This work points to the likely different mechanism of action depending on whether T-705 triphosphate (and its analogue T-1105) are incorporated in place of ATP or GTP. Unfortunately, while the broad-spectrum antiviral activity of T-705 is remarkable in vitro and in vivo, T-705′s strong antiviral effect did not translate into humans during the JIKI Trial against Ebola infections [90] and SARS-CoV-2 clinical trials where T-705 provided only moderate benefits upon early treatment at high doses [89]. One of the major limitations to the use of T-705 in humans is the lower than expected plasma concentration [91,92], including an unanticipated drop in drug concentration observed in patients enrolled in the JIKI Ebola trial [92]. This drop may occur because T-705 possesses a short half-life (2.5–5 h) resulting from rapid renal elimination in the hydroxylated form mediated by aldehyde oxidase [93]. In other words, a major portion of T-705 is eliminated before it gets a chance to be bioactivated to its antiviral form. The fact that T-705 demonstrates a strong antiviral activity against different viruses in a variety of animal models including mice, Guinea pigs, and non-human primates points to the likely human-specific limitations of T-705. Alternatives to T-705 [77,94] and/or strategies to increase its potency are therefore needed.

## 4. Conclusions and Perspectives

The recent and increasing number of viral outbreaks without available treatments underline the critical need to develop broad-spectrum antiviral drugs essential to treat patients rapidly and efficiently. Host-targeted and virus-targeted broad-spectrum antiviral strategies are both needed and likely complementary to offer best possible antiviral coverage and a variety of treatments. Overall, broad-spectrum antiviral drugs are more difficult to design compared to classical virus-specific drugs, due to the differences among viruses, not only in their structures, but also in their behaviors once infecting the host. Indeed, some viruses might require an organ-specific treatment due to the virus tissue tropism while other viruses might require a systemic treatment to reduce viremia. There is a discrepancy between the need of broad-spectrum drug treatments and the antiviral drug research pattern that favors virus-specific research. Antiviral drug research most often uses the logical approach, one virus, one target, one drug (broadly active or not) to clearly understand how a drug is working against a specific virus. Innovation at multiple levels are required to increase discovery of broad-spectrum antiviral drugs, such as the discovery of novel biological mechanisms shared by multiple viruses and the design of medicinal chemistry approaches to target these mechanisms. Additionally, even the most active broad-spectrum antiviral drugs display a range of antiviral activities depending on the virus. Therefore, studying drug combinations to limit drug resistance development and to increase antiviral efficacy is also needed.

## Figures and Tables

**Table 1:** Pros and cons of different antiviral strategies, virus-specific antiviral strategies, host-targeted and virus-targeted broad-spectrum antiviral strategies.

Virus-Specific Antiviral Strategies	Broad-Spectrum Antiviral Strategies	
	Host-Targeted	Virus-Targeted
Pros: - Proven efficacy - Easier design, one viral target - Relative safety compared to other strategies	Pros: - Host proteins broadly required by viruses - Demonstrated antiviral effect - Higher barrier to drug resistance development	Pros: - Less potential for toxicity compared to host-targeted strategies. - Potential for repurposing
Cons: - Narrow application - Low barrier to drug resistance development - Long development time	Cons: - Not selective - Potential for toxicity	Cons: - More complex design - Limited examples of broad-spectrum antiviral drugs

## Data Availability

Not applicable.
